# Retraction Note: Mitochondrial aldehyde dehydrogenase (ALDH2) protects against streptozotocin-induced diabetic cardiomyopathy: role of GSK3β and mitochondrial function

**DOI:** 10.1186/s12916-022-02455-5

**Published:** 2022-07-08

**Authors:** Yingmei Zhang, Sara A. Babcock, Nan Hu, Jacalyn R. Maris, Haichang Wang, Jun Ren

**Affiliations:** 1 Department of Cardiology, Xijing Hospital, Fourth Military Medical University, Xi’an, 710032 China; 2 Center for Cardiovascular Research and Alternative Medicine, University of Wyoming College of Health Sciences, Laramie, WY 82071 USA

**Retraction Note: BMC Med 10, 40 (2012)**

**https://doi.org/10.1186/1741-7015-10-40**

The Chief Editor has retracted this article following an institutional investigation by the University of Wyoming due to concerns regarding data irregularities inconsistent with published conclusions.

Specifically, the investigation found evidence of data irregularities and image reuse in Fig. 7K with previously published articles: Fig. 5 in [1], Fig. 9 in [2], Fig. 6 in [3] and Fig. 3 in [4].

Author Sara Babcock agrees to this retraction. Authors Jun Ren and Yingmei Zhang do not agree to this retraction. Author Haichang Wang has not responded to any correspondence from the editor or publisher about this retraction. The editor was not able to obtain a current email address for Authors Nan Hu and Jacalyn Maris.
